# Metabolic and bariatric surgery reduces the risk of hematological cancer in individuals with obesity: a nationwide administrative data study in France

**DOI:** 10.1097/JS9.0000000000002870

**Published:** 2025-06-27

**Authors:** Julie Bulsei, Sergio Carandina, Viola Zulian, Eric Fontas, Antonio Iannelli

**Affiliations:** a Department of Clinical Research and Innovation, Centre Hospitalier Universitaire de Nice; b ELSAN, Clinique Saint Michel, Centre de Chirurgie de l’Obésité (CCO), Toulon, France; c Université Côte d’Azur, Nice, France; d Adipocible Research Study Group (Université Nice Côte d’Azur and Initiative d’Excellence - Idex), Nice

**Keywords:** hematological cancer, Hodgkin, leukemia, lymphoma, metabolic and bariatric surgery, morbid, multiple myeloma, non-Hodgkin, obesity, Roux-en-Y gastric bypass, sleeve gastrectomy

## Abstract

**Objectives:**

This study aimed to assess the association between Metabolic and Bariatric Surgery (MBS) and the risk of developing hematological cancers in individuals with obesity.

**Summary background data:**

Epidemiological evidence indicates that MBS is associated with a reduction in the risk of solid cancers in individuals with obesity while its effect against the risk of hematological cancer is less clear.

**Methods:**

We conducted a cohort study comparing the risk to develop hematological cancer in individuals with a principal diagnosis of obesity with and without history of MBS that were extracted from the French national hospital discharge database. The protective effect of MBS on the risk of hematological cancer was studied in the overall population and in subgroup analyses according to gender and type of surgical procedure (sleeve gastrectomy vs Roux-en-Y gastric bypass).

**Results:**

The main analysis showed a significant reduction in the risk of Hodgkin lymphoma (0.511 [0.332–0.787]), non-Hodgkin lymphomas (0.467 [0.369–0.591]), leukemia (0.563 [0.452–0.702]) and multiple myeloma (0.389 [0.268–0.566]) in the MBS group after adjusting for age, sex, obesity severity and risk factors for hematological cancer. The propensity score analysis (139 414 individuals in the MBS group versus 278 828 in the control group) confirmed these results. The protective effect of MBS was consistent across genders and type of surgery

**Conclusion:**

MBS is associated with a significant reduction in the risk of hematological cancer in men and women and sleeve gastrectomy and Roux-en-Y gastric bypass are equally effective.

## Introduction

While the casual association between obesity and metabolic disorders including type 2 diabetes, hypertension, dyslipidemia, and increased cardiovascular risk is clearly established, the effect of obesity on the risk of cancer are more heterogeneous. From an epidemiological stand point, there is sufficient evidence associating obesity, defined on the basis of body mass index (BMI), with 13 cancers in humans including cancers of the colon, esophagus (adenocarcinoma), kidney (renal-cell), breast (postmenopausal), corpus uteri, gastric cardia, liver, gallbladder, pancreas, ovary, and thyroid, as well as multiple myeloma and meningioma[1]. On the other hand, in the case of lung cancer individual with obesity not only show a significantly lower risk of developing this cancer but they also show increased survival after curative surgery when compared with lean individuals[2]. In other cases, such as hematological malignancies, evidence is considered convincing only for multiple myeloma,[1] while there is still no consensus for other hematological cancers.

In the last two decades a large body of evidence has been gathered demonstrating that metabolic and bariatric surgery (MBS) is associated with a sustained weight loss and improvement of metabolic conditions, reduced overall mortality, and improved quality of life.^[3-6]^ While most research on the topic has focused on solid tumors, indicating that MBS plays a protective role against the development of certain types of cancers and reduces cancer-related mortality,[4] evidence on the effects of MBS on hematological cancer remains limited.

In the present study, we used the French administrative healthcare database, which is nationwide, among the largest worldwide, and covers 99% of the French population[7], to investigate the hypothesis that MBS is associated with a significant reduction in the risk of hematological cancer. We also aimed at exploring separately the effect of the two most performed MBS procedures (Roux-en-Y gastric bypass (RYGB) and Sleeve Gastrectomy (SG)) and whether the protective effect against cancer risk is consistent across gender. The present study is compliant with the 2025 Transparency in the Reporting of Artificial Intelligence guideline (TITAN)[8].

## Material and methods

### Study design

This study is a retrospective, descriptive, observational study comparing the risk of hematological cancer during follow-up of individual with morbid obesity with (MBS group) or without (control group) a previous history of MBS.

Data were extracted from the French national hospital discharge database (“Programme De Médicalisation des Systèmes d’Information,” PMSI), an exhaustive database on all reimbursed hospital stay in France. The PMSI database contains: patients data (gender, age, etc.), hospital stays data (classification of hospitalizations in diagnosis related groups (DRG), length of stay, etc.), primary and associated diagnoses based on the International Classification of Disease, 10th edition (ICD-10), therapeutic procedures based on the Common Classification of Medical Acts (Classification Commune des Actes Médicaux, CCAM, 11th edition) and economic data (cost of hospital stay, cost of supplements, etc.). Each patient in the database is identified by a unique identifier, which makes it possible to track the patient’s healthcare consumption over time. Due to the anonymous nature of the data, patient consent is not required. The study has been reported in line with the Standards for Quality Improvement Reporting Excellence (SQUIRE) criteria[9]^.^

### Study population

We included all individuals 18 years of age and older with a diagnosis of obesity, i.e., a body mass index (BMI) ≥30 kg/m^2^, based on the ICD-10 and coded during a hospital stay between 1 January 2016 and 31 December 2020. Follow-up ended on the 31 December 2023, or the date of death, whichever occurred first. Were included in the MBS group, individuals who underwent open or laparoscopic RYGB and SG, identified through the CCAM. We excluded individuals with a history of hematological cancer at baseline and within 1 year of inclusion to avoid misdiagnosis during the preoperative MBS assessment. Since the PMSI database covers a 10-year rolling period from 2014 to 2023, a 2-year history (2014–2015) was used to approximate the completeness of baseline risk factors, including prior chemotherapy, radiotherapy and/or brachytherapy, HIV, Down syndrome, and combined immunodeficiency. Finally, individuals in the MBS group were never included in the control group during their pre-MBS period.

### Main analysis

The protective effect of MBS on the risk of hematological cancer was studied in the overall population. Hematological cancer were identified through the ‘C81-C96ʹ ICD-10 group of “Primary or presumed primary malignant tumors of lymphoid, hematopoietic and related tissues” and have been divided into 6 major groups: Hodgkin’s lymphoma; non-Hodgkin’s lymphoma; malignant immunoproliferative diseases; multiple myeloma and malignant plasma cell tumors; leukemia; malignant tumors of lymphoid, hematopoietic and related tissues, other and unspecified.

**HIGHLIGHTS**

- This is the first large-scale national study to demonstrate a protective effect of bariatric surgery against four major hematological cancers: Hodgkin lymphoma, non-Hodgkin lymphoma, leukemia, and multiple myeloma.
- The study provides new comparative data showing that both sleeve gastrectomy and Roux-en-Y gastric bypass are equally effective in reducing hematological cancer risk.
- The protective association is consistent across both men and women, challenging prior evidence suggesting a gender-specific benefit.
- Findings suggest that metabolic improvements and weight loss from MBS may reduce hematopoietic cancer risk, expanding the recognized benefits of bariatric surgery beyond solid tumors.
- These results support considering MBS as a preventive strategy not only for metabolic disease but also for certain blood cancers in individuals with obesity.

### Sub-group analyses

Sub-group analyses were performed to assess the protective effect of MBS against the risk of hematological cancer according to the type of procedure (RYGB vs SG) and gender (men vs women). We performed these analyses according to procedure type, as SG and RYGB have never been compared in the literature, and according to gender, as current data indicate that MBS is protective only in women[10].

### Statistical analysis

Patients’ characteristics in the two groups were described by mean (standard deviation) for quantitative data and frequency (percentage) for qualitative data and compared using respectively the Student’s t test and the Chi2 test or Fisher’s exact test.

The analyzed covariates were: demographic: age, gender, obesity severity (stratified as follows in the PMSI database: BMI from 30 to 39.9 kg/m^2^, 40 to 50 kg/m^2^ and >50 kg/m^2^); risk factors for hematological cancers: history of previous chemotherapy, radiotherapy and/or brachytherapy; HIV infection; Down syndrome; combined immunodeficiency.

The risk of hematological cancer was expressed as the number of new cases per person-year of follow-up (PYFU) and the association between group and risk of hematological cancer was studied using a Cox model adjusted on age, sex, obesity severity and comorbidities.

In order to assess the uncertainty around the results, a 1:2 propensity score matching was performed using the nearest neighbor method, with a 0.5 caliper, to create two groups of comparable patients. The propensity score was estimated with a logistic regression model using age, sex, obesity severity and risk factors for hematological cancer as covariates. Kaplan–Meier’s curves were plotted from the matched populations and then the association between groups and risk of hematological cancer was studied using a univariate Cox model.

All tests were two-sided and *P* values <0.05 were considered statistically significant. We used SAS Enterprise Guide software version 7.1 (SAS institute, Inc, Cary, North Carolina, USA) for statistical analyses.

## Results

### Overall population analysis

**Figure 1.**
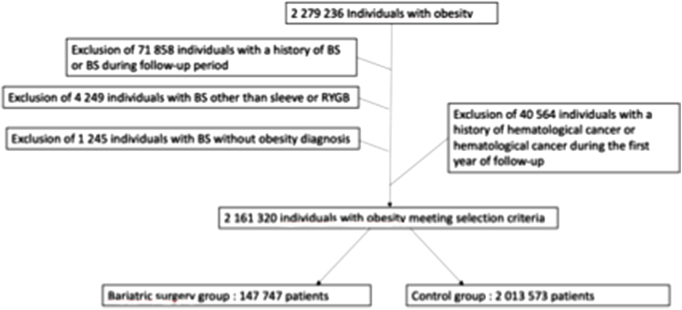
Flow chart.

Legend: BS, bariatric surgery; RYGB, Roux-en-Y gastric Bypass; SG, sleeve gastrectomy.

Among the 2 279 236 individuals with a diagnosis of obesity in the general French population, we identified 147 747 individuals with a history of MBS (MBS group) and 2 013 573 individuals with a diagnosis of obesity and no history of MBS (control group). Of them 4249 individuals did not meet the inclusion criteria (procedure type) and 113 667 were excluded (previous history of MBS or hematological cancer or MBS in the absence of a primary diagnosis of obesity) (Fig. 1). The average follow-up was 5.4 years (SD 1.2) in the MBS group and 5.8 years (SD 1.4) in the control group (*P* < 0.0001). As expected, the mean age was significantly higher in the control group (59.1 (SD 18.0) vs 41.0 (SD 12.1) years) and there were more women and more individuals with massive obesity in the MBS group, while risk factors for hematological cancers were higher in the control group. The large majority of individual in the MBS group had a SG (108 071 patients (73.2 %)) (Table 1).

**Table 1:** Characteristics at baseline in the overall population

Patients characteristics	BS N = 147 747	Control N = 2 013 573	*P* value
Age, mean (SD)	41.0 (12.1)	59.1 (18.0)	<0.0001
Gender, men frequency (%)	31 925 (21.6)	854 287 (42.4)	<0.0001
Obesity severity, frequency (%)			
30 to 40 kg/m2	52 749 (35.7)	1 645 969 (81.7)	<0.0001
40 to 50 kg/m2	80 759 (54.7)	317 802 (15.8)	
> 50 kg/m2	14 239 (9.6)	49 802 (2.5)	
Risk factors for HC (%)			
History of chemotherapy	2453 (1.7)	127 393 (6.3)	<0.0001
History of radiotherapy	234 (0.2)	30 717 (1.5)	<0.0001
History of brachytherapy	24 (0.0)	1416 (0.1)	<0.0001
History of HIV	245 (0.2)	4650 (0.2)	<0.0001
History of Down syndrome	7 (0.0)	1034 (0.1)	<0.0001
History of combined immunodeficiency	2 (0.0)	151 (0.0)	0.0033
Procedure type, frequency (%)			
RYGB	39 676 (26.8)	NA	NA
SG	108 071 (73.2)	NA	

BS, bariatric surgery; HC, hematological cancer; HIV, human immunodeficiency virus; RYGB, Roux-en-Y gastric bypass; SD, standard deviation; SG, sleeve gastrectomy.

The number and the crude rate of hematological cancers per 1000 individuals PYFU in each group are presented in Table 2. Leukemia, non-Hodgkin’s lymphomas and multiple myeloma were the most common hematological cancers in the whole study population.

**Table 2:** Number and crude rate of hematological cancers in the BS group versus the control group

	BS N = 147 747	Control N = 2 013 573
	Number of hematological cancers (frequency (%))	
Hodgkin’s lymphoma	23 (0.02)	660 (0.03)
Non-Hodgkin’s lymphomas	74 (0.05)	4769 (0.23)
Malignant immunoproliferative diseases	16 (0.01)	923 (0.05)
Multiple myeloma and malignant plasma cell tumors	29 (0.02)	2518 (0.12)
Leukemias	85 (0.06)	5464 (0.27)
Malignant tumors of lymphoid, hematopoietic and related tissues, other and unspecified	7 (0.00)	245 (0.01)
	Crude rate of hematological cancers (per 1000 individuals PYFU [95 CI])	
Hodgkin’s lymphoma	0.029 [0.029; 0.029]	0.062 [0.062; 0.062]
Non-Hodgkin’s lymphomas	0.093 [0.093; 0.094]	0.448 [0.448; 0.448]
Malignant immunoproliferative diseases	0.020 [0.020; 0.020]	0.086 [0.086; 0.086]
Multiple myeloma and malignant plasma cell tumors	0.036 [0.036; 0.037]	0.236 [0.236; 0.236]
Leukemias	0.107 [0.106; 0.108]	0.513 [0.513; 0.514]
Malignant tumors of lymphoid, hematopoietic and related tissues, other and unspecified	0.009 [0.009; 0.009]	0.023 [0.023; 0.023]

BS, bariatric surgery.

The main analysis showed a significant reduction in the risk of hematological cancers for Hodgkin lymphoma (0.511 [0.332–0.787]), non-Hodgkin lymphomas (0.467 [0.369–0.591]), leukemia (0.563 [0.452–0.702]) and multiple myeloma (0.389 [0.268–0.566]) in individuals with obesity in the MBS group compared to those in the control group after adjusting for age, sex, obesity severity and risk factors for hematological cancer. This risk reduction was not observed for malignant immunoproliferative diseases and malignant tumors of lymphoid, hematopoietic and related tissues.

**Figure 2.**
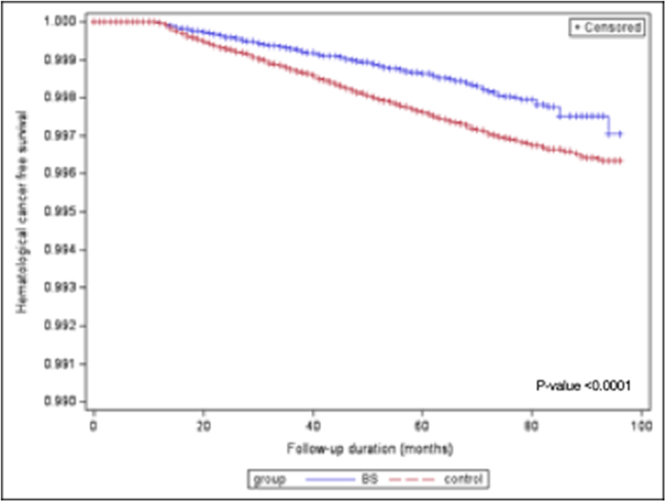
Kaplan–Meier’s curves after sensitivity analysis for all hematological cancers over time. Legend: BS, bariatric surgery.

**Figure 3.**
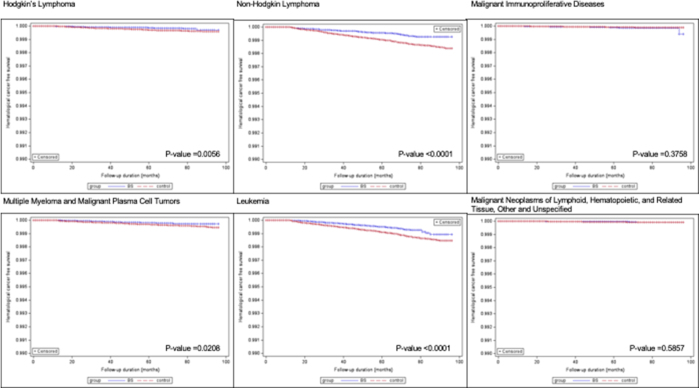
Kaplan–Meier’s curves after sensitivity analysis for each hematological cancer over time. Legend: BS, bariatric surgery.

The results of the sensitivity analysis, including 139 414 individuals in the MBS group versus 278 828 in the control group, confirmed the results obtained in the main analysis although the protective effect of MBS against the risk of multiple myeloma was mitigated as the HR increased up to 0.613 [0.405; 0.928] (Table 3). Kaplan–Meier’s curves after sensitivity analysis, presented in Fig. 2, show the protective effect of MBS against the risk of all hematological cancers over time. Figure 3 presents Kaplan–Meier’s curves after sensitivity analysis for each hematological cancer.

**Table 3:** Risk of hematological cancer during follow-up in the BS group versus the control group

Group (ref = control)	HR [95% CI]	*P* value
Main analysis		
Hodgkin’s lymphoma	0.511 [0.332; 0.787]	0.0023
Non-Hodgkin’s lymphomas	0.467 [0.369; 0.591]	<0.0001
Malignant immunoproliferative diseases	0.758 [0.454; 1.264]	0.2883
Multiple myeloma and malignant plasma cell tumors	0.389 [0.268; 0.566]	<0.0001
Leukemias	0.563 [0.452; 0.702]	<0.0001
Malignant tumors of lymphoid, hematopoietic and related tissues, other and unspecified	0.567 [0.260; 1.235]	0.1530
Sensitivity analysis		
Hodgkin’s lymphoma	0.508 [0.315; 0.820]	0.0056
Non-Hodgkin’s lymphomas	0.492 [0.381; 0.636]	<0.0001
Malignant immunoproliferative diseases	1.329 [0.708; 2.496]	0.3758
Multiple myeloma and malignant plasma cell tumors	0.613 [0.405; 0.928]	0.0208
Leukemias	0.595 [0.466; 0.760]	<0.0001
Malignant tumors of lymphoid, hematopoietic and related tissues, other and unspecified	0.784 [0.327; 1.879]	0.5857
Men’s subgroup		
Hodgkin’s lymphoma	0.340 [0.138; 0.838]	0.0191
Non-Hodgkin’s lymphomas	0.486 [0.330; 0.716]	0.0003
Malignant immunoproliferative diseases	0.986 [0.479; 2.027]	0.9691
Multiple myeloma and malignant plasma cell tumors	0.458 [0.257; 0.816]	0.0081
Leukemias	0.694 [0.498; 0.967]	0.0311
Malignant tumors of lymphoid, hematopoietic and related tissues, other and unspecified	0.300 [0.041; 2.199]	0.2361
Women’s subgroup		
Hodgkin’s lymphoma	0.600 [0.365; 0.986]	0.0439
Non-Hodgkin’s lymphomas	0.462 [0.343; 0.623]	<0.0001
Malignant immunoproliferative diseases	0.623 [0.302; 1.284]	0.1994
Multiple myeloma and malignant plasma cell tumors	0.358 [0.220; 0.585]	<0.0001
Leukemias	0.484 [0.361; 0.650]	<0.0001
Malignant tumors of lymphoid, hematopoietic and related tissues, other and unspecified	0.641 [0.270; 1.518]	0.3117
RYGB subgroup		
Hodgkin’s lymphoma	0.256 [0.082; 0.798]	0.0189
Non-Hodgkin’s lymphomas	0.530 [0.354; 0.795]	0.0021
Malignant immunoproliferative diseases	0.162 [0.023; 1.158]	0.0697
Multiple myeloma and malignant plasma cell tumors	0.373 [0.185; 0.749]	0.0055
Leukemias	0.529 [0.350; 0.799]	0.0025
Malignant tumors of lymphoid, hematopoietic and related tissues, other and unspecified	1.210 [0.441; 3.317]	0.7110
SG subgroup		
Hodgkin’s lymphoma	0.602 [0.380; 0.954]	0.0308
Non-Hodgkin’s lymphomas	0.439 [0.331; 0.583]	<0.0001
Malignant immunoproliferative diseases	1.000 [0.590; 1.694]	0.9991
Multiple myeloma and malignant plasma cell tumors	0.393 [0.254; 0.609]	<0.0001
Leukemias	0.578 [0.447; 0.746]	<0.0001
Malignant tumors of lymphoid, hematopoietic and related tissues, other and unspecified	0.333 [0.105; 1.059]	0.0625

RYGB, Roux-en-Y gastric bypass; SG, sleeve gastrectomy.

### Sub-group analyses

Interestingly, the results of the sub-group analyses were in line with those of the main analysis indicating that both SG and RYGB were equally effective and both in women and men against the risk of hematological cancer (Table 3). Men and women Kaplan–Meier’s curves, after propensity score matching, are presented in Supplementary Digital Content, Annex 1 (available at: http://links.lww.com/JS9/E550).

## Discussion

This study indicates that MBS is associated with a reduced risk of hematological cancer including Hodgkin and non-Hodgkin lymphomas, leukemia and multiple myeloma.

One of the emerging areas of interest in oncology is the impact of weight loss and particularly through MBS on cancer risk, as obesity is a recognized risk factor for at least 13 cancers. The specific case of hematological cancer stands apart as only multiple myeloma among hematological cancers is considered an obesity-related cancer. However, there is growing evidence that obesity is a risk factor for other hematological cancers including leukemias and lymphomas.

There is a body of epidemiological evidence including large cohort studies and meta-analyses of cohort and case control studies indicating a significantly elevated risk for hematological cancer in people with an elevated BMI[11]. Furthermore, as BMI has several limitations as a marker of obesity, because it does not include any information on adipose distribution and visceral fat has been linked to several worse health outcomes^[12,13]^, it may be speculated that the association between specific subtypes of obesity and hematological cancer is even stronger. Although, the epidemiological evidence of the association between obesity and hematological cancer seems to be established, the mechanisms underlying this association remain to be largely elucidated. It is well established that obesity is responsible for metabolic, endocrinologic, immunologic, and inflammatory-like changes that may favor cancer development that may act increasing cell mutation rate, dysregulating gene function, interfering with DNA repair, but also through epigenetic changes[14]. The 2017 CDC Vital Signs report by Steele *et al* highlighted that 55% of cancers diagnosed in postmenopausal women and 42% of all cancers in the USA are linked to obesity, emphasizing the critical need for weight management strategies in cancer prevention[15] Importantly, several studies have shown that MBS-induced weight loss significantly reduces cancer incidence. Schauer *et al* provided robust evidence supporting this relationship, demonstrating a lower risk of cancer following substantial weight loss after MBS[4]. Similarly, findings from the Longitudinal Assessment of Bariatric Surgery consortium cancer risk is reduced >50% when weight loss after MBS, exceeds 20% TWL compared with patients with <20% TWL. These data reinforce the role of bariatric surgery not only in managing obesity-related comorbidities but also as a key intervention in cancer prevention[16].

Emerging data suggest that MBS may also reduce the risk of hematological cancers. Rustgi *et al* showed in a retrospective cohort study that MBS reduces the risk of any cancer and obesity-associated cancers, including multiple myeloma (MM), in individuals with non-alcoholic liver disease[17]. Tao *et al* reported a reduced risk of non-Hodgkin lymphoma in women in a retrospective study based on national patient registries in all Nordic countries (Denmark, Finland, Iceland, Norway and Sweden) from 1980 to 2012[18]. More recently, Sjöholm *et al* reported evidence from the prospective Swedish Obese Subjects study indicating that MBS is associated with a reduced incidence of hematological cancers and reduced mortality in women[10]. While our study provides robust epidemiological evidence suggesting a protective association between metabolic and bariatric surgery (MBS) and the incidence of hematological malignancies, it does not allow us to explore the underlying biological mechanisms. It is plausible that improvements in metabolic parameters following MBS – such as reductions in insulin resistance, hyperglycemia, chronic low-grade inflammation, and adipokine dysregulation – contribute to mitigating oncogenic processes in hematopoietic cells. These factors have been implicated in clonal hematopoiesis, immune dysfunction, and increased oxidative stress, all of which may promote hematological carcinogenesis^[11,19]^. However, further translational and clinical research is essential to elucidate how MBS may influence the pathways involved in hematopoietic oncogenesis.

A main feature of our study is the evidence that MBS is associated with a significant reduction in the risk of MBS for the four main hematological cancers, namely Hodgkin and non-Hodgkin lymphomas, leukemia and multiple myelomas. Although we provide only epidemiological evidence, the homogeneity of the association (size and direction) between MBS and the risk of hematological cancer across the four cancer categories add strength to our results.

Our data indicate MBS is equally effective in reducing the risk of hematological cancers in both men and women. This finding stands in contrast to the findings of Sjöholm *et al*, who reported a greater reduction in hematological cancer risk among women compared to men following MBS[10]. Several important methodological and clinical distinctions may account for the discrepancies in findings regarding the effect of gender. In our cohort, the proportion of men in the MBS group was approximately 20% and nearly double in the control group. However, our cohort included a substantially larger number of individuals than the SOS study, which may have provided sufficient statistical power to observe a protective effect of MBS in men, effectively eliminating the gender difference seen in the Swedish cohort. In addition, the types of surgical procedures performed differ significantly between the two studies. The SOS cohort mainly involved gastric banding and vertical banded gastroplasty – procedures that are now largely obsolete – whereas our study included contemporary and more effective techniques, such as sleeve gastrectomy (SG) and Roux-en-Y gastric bypass (RYGB)[20]. These procedural differences may have distinct metabolic, inflammatory, and immunological impacts, potentially influencing cancer risk and outcomes differentially across sexes. Another important distinction lies in the control group characteristics. Our control group did not receive structured lifestyle support or long-term follow-up, unlike the SOS control cohort, which was monitored and encouraged to adopt healthier behaviors over time. This discrepancy may have amplified the relative benefit of MBS in our study, particularly among men, who are often less engaged in routine preventive care. Finally, it is important to note that the median follow-up duration in the SOS study was nearly 30 years, whereas the follow-up period in our study was significantly shorter. This difference in follow-up time may influence the timing and detection of cancer events, and possibly the observed impact of MBS across subgroups. Additionally, MBS has evolved substantially since the early phases of the SOS cohort. Advances in surgical technique, perioperative care, and patient selection – including the increased inclusion of men and individuals at higher surgical risk – may have contributed to narrowing historical sex-based differences in outcomes. These combined differences in population characteristics, procedures, control group management, sample size, and follow-up duration likely explain the divergence in findings between the two studies.

The absence of a significant protective effect of MBS on malignant immunoproliferative diseases and other unclassified tumors in our study is likely explained by two main factors. First, the number of cases in both groups was extremely low: only 7 cases out of 147 757 individuals in the MBS group and 245 cases out of over 2 million individuals in the control group. This very small number limits the statistical power to detect meaningful differences between groups. Second, this diagnostic category is inherently heterogeneous, encompassing a broad spectrum of rare and poorly defined entities, which may differ significantly in etiology, risk factors, and pathophysiology. This heterogeneity makes it difficult to draw robust conclusions regarding the impact of MBS on this group as a whole.

We also found that that there was no difference in the effectiveness of SG and RYGB in reducing the risk of hematological cancers. This is a new finding as these two procedures, which are currently the most commonly performed, had not been compared before with respect to their protective effect against this specific cancer. This probably indicates that the improvements in metabolic health, inflammation, and immune function following either surgery are linked mainly to the loss of weight and may play a more crucial role in cancer risk reduction than the specific surgical technique used.

Sjöholm *et al* pointed out at a reduced mortality in individuals with a hematological cancer and a previous history of MBS compared to those with a hematological cancer in control group who had no MBS in the prospective controlled SOS cohort[10]. The authors speculated that individuals with obesity may be an aggravating factor leading to a poorer prognosis. However, Haddad *et al* recently compared in a retrospective study 22 patients identified out of a 652 with chronic myeloid leukemia to a matched cohort of 44 patients with no prior MBS, and found, that in multivariate analysis MBS was the only independent predictor for the risk of treatment failure or event-free survival[20]. While the main finding was the association between MBS history and oral tyrosine kinase inhibitors treatment failure, the authors also found a lower rate of early molecular remission and the slower response dynamics which are main predictors of outcome in chronic myeloid leukemia[22]. Several factors may be claimed as responsible for the decreased bioavailability of tyrosine kinase inhibitors including a decreased absorption related to the altered gastric emptying and accelerated intestinal transit related to the altered anatomy due to MBS and the use of proton pomp inhibitors, which is very common after MBS. These findings, although retrospective and in a limited monocentric series, prompt the need to design specifically adapted treatment strategies for these patients^.[22]^

Our study carries several limitations inherent to its retrospective design and reliance on administrative data. As with all retrospective analyses, causality cannot be firmly established, and residual confounding may persist despite robust matching methods. Notably, the PMSI database does not provide information on weight trajectories or changes in BMI over time, nor does it allow for the assessment of cancer-related mortality. Additionally, we were unable to account for the impact of lifestyle interventions such as dietary counseling or physical activity, which may influence cancer risk either independently or in synergy with metabolic and bariatric surgery. These limitations should be considered when interpreting the results and underscore the need for prospective studies with detailed clinical and behavioral data. Furthermore, while the accuracy of diagnosis coding in administrative databases is a potential source of bias, obesity in the PMSI is typically coded based on data from clinical records that include actual anthropometric measurements (e.g., BMI assessed during hospitalization), rather than self-report, which increases reliability. Furthermore, while our propensity score matching approach aimed to reduce baseline differences between groups, unmeasured confounders – such as health-seeking behavior and differential access to diagnostic workup – may still have contributed to the reduced incidence of hematological malignancies observed in the MBS group. However, the PMSI stands among the largest healthcare databases worldwide, and is considered reliable due to several key factors. First, it is a comprehensive and mandatory reporting system, which ensures that all hospital admissions across France are consistently recorded, creating a large and robust dataset. The database covers a wide range of healthcare facilities, from public hospitals to private clinics, providing a national scope of health data. Additionally, the PMSI is subject to strict regulations and standardized coding practices, including the use of international classifications like ICD-10, ensuring consistency and accuracy in the recording of diagnoses and procedures. Additionally, the PMSI is regularly audited and subjected to data validation processes to enhance accuracy and reliability. Importantly, it also serves as the basis for hospital reimbursement in France, meaning that hospitals are incentivized to report their data accurately, as their financial compensation is directly tied to the information submitted. This further ensures the quality and completeness of the database, making it a valuable resource for healthcare research, policy development, and epidemiological studies.

In conclusion, the present study provides robust evidence that in individuals with obesity MBS is associated with a significant reduction in the risk of Hodgkin and non-Hodgkin lymphomas, leukemia and multiple myeloma compared to those with no history of MBS.

## Data Availability

Data cannot be shared publicly because access to the PMSI database (French Medical Information System Program) is restricted to authorized professionals with individual access. The collected data are confidential, and only processed data can be disseminated as demonstrated in our work. To access raw data, an authorized French professional must be contacted to access the PMSI or the institution must request access to the database by contacting: demande-base@atih.sante.fr.

## Research registration unique identifying number (UIN)

Retrospective study using data from the French national hospital discharge database.
